# Effects of Electroencephalogram Neurofeedback Intervention on Blood C-Reactive Protein Levels in Astronauts Attending 2-Week Long Analog Moon Mission

**DOI:** 10.3390/brainsci14080843

**Published:** 2024-08-22

**Authors:** Jakub Hinca, Marcin Dornowski

**Affiliations:** Department of Physical Culture, Gdansk University of Physical Education and Sport, 80-336 Gdansk, Poland; marcin.dornowski@awf.gda.pl

**Keywords:** stress, isolation, EEG neurofeedback, inflammation

## Abstract

The human organism is affected by multiple stressors every single day, especially during extremely demanding activities. It needs a method to regulate itself better. One of the stressors that is affecting humans is social isolation. The state of prolonged isolation happens during space missions. In this study, 40 analog astronauts attended a two-week-long mission. The experimental group had EEG neurofeedback training intervention performed on a daily basis, while the control group remained isolated without neurofeedback. The results let us take this non-invasive intervention under consideration, while debating the methods to lower the physiological stress reaction in humans that are exposed to extremely hard circumstances. Although not statistically significant, the trends observed give us direction towards other research to confirm EEG neurofeedback as a method to lower cell stress response levels.

## 1. Introduction

Social isolation (SI) is considered to be a stressor that can lead to many health issues, such as cardiovascular diseases [1], mental health problems [2], or even raised blood C-reactive protein (CRP) levels [3]. Furthermore, the loneliness that is linked with a state of SI is considered to be a factor affecting human behavior, such as physical activity [4]. Isolation can be caused by environmental or occupational factors. It is also important for older adults to participate socially as it relates to enhancing subjective well-being [5]. When it comes to the occupational side of the problem, some jobs, such as astronauts or ground-based space missions, include living in a prolonged state of isolation [6]. Many health conditions are connected with an increase in blood C-reactive protein levels, as it refers to the inflammation process happening in the cells [7]. The release of CRP also happens during an exposition to certain stressors (e.g., temperature or physical activity to exertion) [8]. Some studies also suggest that working in a state of prolonged social isolation may increase the risk of cognitive impairments, sleep disturbance, lower job-related satisfaction and mood [9]. The CRP level is likely more stable than the cortisol levels during the day. This is because of the circadian rhythm that has an impact on hormone release [10]. The C-reactive protein is a molecule that is secreted in liver, mainly by hepatocytes. Its levels increase as a response to an inflammatory process. The CRP is known as an “acute phase protein” as it is also connected with tissue injury [11]. This was discovered in 1930 by Tillet and Francis and happened during the research on Pneumococcus infection in patients [12]. This molecule helps an organism to clear itself of dead or dying cells. It is made by the binding of the CRP to the phosphocholine, which is expressed in dead or dying cells. This binding works as a marker for phagocytosis cells to neutralize them [13]. The C-reactive protein also stimulates the complement system, which raises the ability of these cells to clear microbes and the cells that have been damaged [14]. The CRP is clinically relevant as a marker of inflammation, which is happening in many medical conditions. Even though it is non-specific, elevated CRP levels in humans suggest that an inflammatory process is happening somewhere in the body. In the case of need, it is also possible to measure the level of the hs-CRP (high-sensitivity CRP) that is more specific and is used as a diagnostic tool to assess cardiovascular disease risk [15]. This was also observed in elderly people in chronic social isolation. Social isolation is considered to be a significant stressor in the long-term, which means it can be investigated in terms of the coexistent CRP release during isolation. Psychological stress response to SI can be modulated through HPA axis over-arousal because of chronic stress. Cortisol has anti-inflammatory properties, which means that disbalance in HPA axis activation can lead to a dysregulation of the immune response and an increased production of the pro-inflammatory cytokines [16].

For decades, our society has worked on effective space exploration. Because of the numerous benefits of interacting with the Earth from the level of space [17], many nations seek a path to discover the limitations which could affect the efficacy of a human presence in space. Analog space missions seem to be a partial solution to this problem. They are made to simulate the challenges that a human organism meets both physically and mentally, such as social isolation, simulated microgravity and emergency situations. They provide educational projects for primary schools, secondary schools and students, as the space exploration topic becomes more and more popular, and it is a real catalyst for the future space colonization process [18]. These kinds of missions can be completed both in an educational matter and in a scientific matter.

One of the many methods that allow us to decrease the level of stress and also the high CRP level is mindfulness relaxation [19], but another kind of method called EEG neurofeedback seems to be even more perspective towards the goal of lowering the stress response in humans.

EEG neurofeedback uses facts known about the electrophysiology of the brain in treating many disorders with training focused on activation and re-establishment of brain electrophysiology. Its effectiveness is seen in attention deficiency disorders [20], anxiety-related stress [21] and post-traumatic stress disorder [22], as mentioned conditions seem to generate symptoms that are connected to over- or under-arousal of particular regions of the human brain. Another recent finding includes lowering stress-related response in young males after using the EEG neurofeedback training protocol [8].

The aim of this study is to determine if the daily EEG biofeedback training sessions performed by astronauts were associated with lowering the cell stress response in the 14-week-long analog space mission.

## 2. Materials and Methods

### 2.1. Participants

The participants were healthy male (20) and female (20) analog astronauts (taking part in analog missions in Habitat Lunares Research Station in PIła, Poland). Each of the subjects expressed their consent to participate in the research with a letter. All study procedures were in accordance with the ethical standards of the bioethical research committee and with the 1964 Helsinki Declaration and its subsequent amendments or comparable ethical standards. All subjects were healthy and without any neurological disorders. Subjects were randomly divided into control (isolation) and intervention (isolation with EEG neurofeedback) groups. Intervention group age was 26.6 ± 6.08, whereas control was 23.4 ± 2.07. The sex and personal data of the subgroup of participants was unknown to the researchers. The inclusion criteria were qualifying for the group of analog mission members, age 20–25, and an impeccable health record (including parameters that were analyzed during the experiments and confirmed by a doctor).

The exclusion criterion was deterioration of health during the mission, excluding the participant from continuing the mission.

### 2.2. Study Design and Procedure

Blood CRP levels were measured in subjects before the intervention. Later on, each of the participants was exposed to analog space mission isolation for 2 weeks. EEG neurofeedback sessions were implemented on the intervention group each day of the mission. After the isolation, both groups participated in the second measurement—measuring blood CRP levels after the isolation.

### 2.3. EEG Neurofeedback Training Protocol

In the experiment, EEG neurofeedback was used as a diagnostic and intervention method. EEG neurofeedback device is relatively compact and is made of several parts. EEG NF Device used to carry out the protocol was ProComp 5 Infiniti Biomed (Wrocław, Poland). Main features of this system are five input channels, which allow the recording of multiple physiological feedback signals; light weight of the device and its portability, which allows ease of use in many environments; and software, which is connected and integrated with the device and allows real-time visualization and customizable protocols for neurofeedback. These reasons made ProComp 5 Infiniti Biomed suitable for not only this particular research but also for other studies by our team. The bandwidth of the ProComp 5: DC 512 Hz @ 2048 samples/s DC 64 Hz @ 256 samples/s DC 64 Hz @ 200 samples/s DC 8 Hz @ 32 samples/s DC 8 Hz @ 20 samples/s signal sampling rate ranges from 20 sps up to 2048 sps. At first, it is crucial to find anthropometric point at the top of head. It is called vertex. It is found by measuring the distance between inion and nasion—points between the eyes and the external occipital protuberance. In the middle of the line between these two points, one may find the vertex. Afterwards, it is necessary to clean subject’s electrode placement points with dedicated paste with peeling. After performing the scrub, special glue is used to attach electrodes to the vertex and on the side of head. Also, there are two referral electrodes attached to the earlobes. After successfully attaching electrodes—it is necessary to check if the impedance is sufficient to collect data about the bioelectric brain function. Afterwards, there is a protocol card made in software, and it is possible to perform a diagnostic measurement of a particular wave’s frequency and amplitude. In the entire group of subjects (experimental and control), point Cz (vertex) and C3 from the central belt were used in the QEEG point examination. Reduced activity (many slow waves) in the central belt may be associated with difficulties with sensorimotor coordination. Increased activity (a lot of fast waves) may be associated with tics, tremors and obsessive–compulsive behavior. The intervention used the protocol: Cz—alpha strengthening, theta lowering and beta lowering.

During diagnostic session (QEEG), subjects received 4 tasks—having eyes open, having eyes closed, attention focused and making cognitive effort (e.g., counting). Achieved results allowed authors to set individual and proper training protocols for each intervention group astronaut. The training session protocol involved 4 tasks: baseline eyes open, baseline eyes closed, sensory attentiveness and cognitive effort. The waves measured were alpha, beta, theta and SMR waves. Also, the values of theta/alpha and SMR/theta were measured. Each participant executed protocols with 2 EEG points and involved two brain waves. Neurofeedback EEG sessions were conducted via ProComp5 Infiniti Biomed.

### 2.4. Statistical Analysis

Data normality was confirmed by the Shapiro–Wilk test. Data analysis was performed using STATISTICA 10 package, using variation analysis. Descriptive statistics were used throughout the study, and the Student’s *t*-test and two-way ANOVA were used to determine differences between the obtained results.

## 3. Results

During the EEG neurofeedback experiment intervention, almost all the subjects had a decreased factor of the alpha wave (during pre-mission tests) responsible for rest, relaxation and creativity, which may be related to the lack of calmness and rest, and increased beta 1 waves, responsible for the state of wakefulness, thinking and concentration, external orientation, focusing attention and solving problems. The very low alpha wave index in the case of one subject is noteworthy.

In the intervention group (isolation with EEG neurofeedback) a lowered blood CRP level was observed compared to the control group (isolation without EEG neurofeedback).

**Figure 1:**
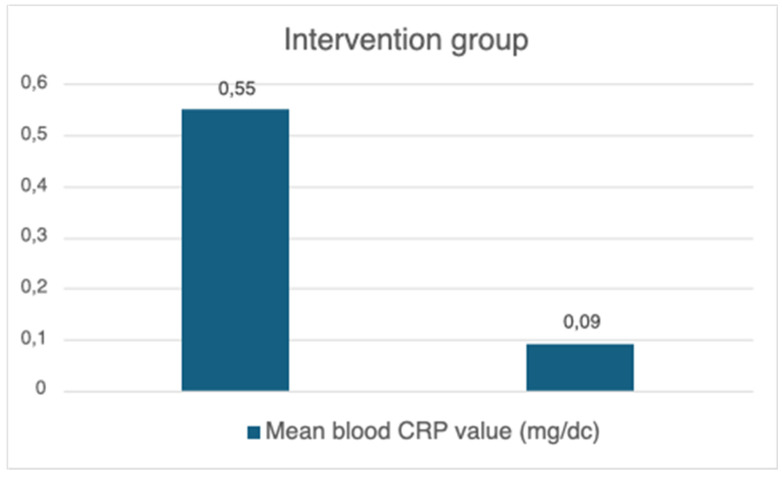
Mean blood CRP value before and after isolation with EEG neurofeedback.

The mean blood CRP level in the intervention group before isolation was 0.55 mg/dc, with the standard deviation (SD) equating to 1.07 (Figure 1). After the isolation, the mean blood CRP level was 0.09 mg/dc, with the standard deviation equating to 0.05. Basic statistics did not show significant changes. However, there were important tendencies which occurred during the experimental process.

**Figure 2:**
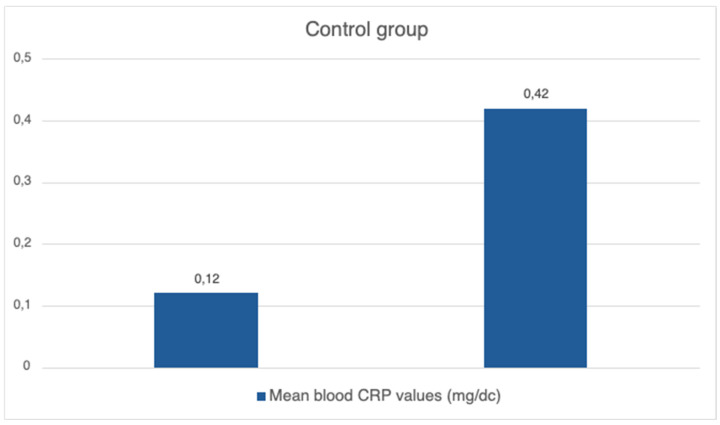
Mean blood CRP value before and after isolation in control group (without EEG neurofeedback).

The mean blood CRP level in the control group before isolation was 0.12 mg/dc, with SD equating to 0.09. After the isolation and intervention, the mean blood CRP level was 0.42 mg/dc, with the standard deviation equating to 0.82 (Figure 2).

The parameters described above appeared to be worse after isolation for the control group, but they were not statistically significant. In the experimental group, the value of the blood CRP level after intervention was lower, but the result was not statistically significant. The normality of the results was measured with the Shapiro–Wilk’s test and the Wilcoxon test.

The statistical tests (t-Student test) have not yet confirmed the observed trends. However, a comparison of the arithmetic means of the results obtained in the CRP blood test indicates the expected direction of changes. The CRP parameters decreased in the experimental group in which EEG biofeedback was used during the analog mission. It is assumed that increasing the research group (experimental and control), thanks to subsequent analog missions, will allow confirmation of these trends with statistical tests. So far, it is possible to call it significant, at the level of statistical tendency. Two-way ANOVA showed the following results: groups: F(1,17) = 0.054, *p* = 0.821; time: F(1,17) = 0.036, *p* = 0.852; interaction between groups: F(1,17) = 6.578, *p* = 0.023. Effect size (eta squared)—group: η2 = 0.0027\eta^2 = 0.0027η2 = 0.0027, time: η2 = 0.0018\eta^2 = 0.0018η2 = 0.0018; interaction between groups: η2 = 0.2477\eta^2 = 0.2477η2 = 0.2477. Group and time have no significant effect on CRP (*p* > 0.05). The interaction between groups and time is significant (*p* = 0.023), suggesting that the effect of time on the CRP varies by group. The interaction effect (eta squared = 0.2477) is significant, meaning that the interaction between groups and time has a relatively large effect compared to the main effects.

## 4. Discussion

A prolonged state of social isolation can be harmful to human health. That is the reason to look for interventions that can be beneficial and that can lower the potential adverse effects of working in space. Additionally, it is possible to provide some additional data using self-report questionnaires about subjective well-being; however, there is also a higher risk of bias due to the inaccurate or dishonest reporting by participants [23]. Remaining in a state of microgravity is linked to numerous adverse health effects since every system of the human organism is exposed to a large amount of stress. That is why astronauts are thoroughly screened in a medical manner before, during and after the spaceflight [24]. Comparing our findings with interventions such as mindfulness meditation, during an EEG neurofeedback session, it is possible to observe particular waves’ amplitude ratios. For example, a high theta wave amplitude is correlated with a state of attention deficit, and a low alpha wave amplitude is correlated with exhaustion and mind fatigue. When it comes to the biochemical markers of stress, the high CRP levels are connected with a state of acute injury, tissue damage, infection or inflammation. A stressful environment can also lead to a rise in cortisol levels [25]. However, we decided not to measure this hormone because of the correlation between releasing it and circadian rhythms [10]. Even though this research shows the positive influence of EEG neurofeedback on the stress response in the analog mission astronauts, further research with more subjects and different training protocols is recommended. A relatively small sample size may limit the generalizability of the current findings. More statistical significancy is needed to establish the statement that EEG neurofeedback intervention is effective for a larger population. It is also advised to conduct a study using a placebo group. Additionally, specific statistics and an approach should be taken under consideration according to the analyzed groups (such as astronauts or athletes—where all of them are on a similar level of diagnostic parameters).

Neurofeedback is a method that is under the process of very fast development and implementation. It gathers interest in research around neurobiopsychology and non-invasive treatments of several conditions such as ADHD, in which there is a tendency for personalized treatment protocol to be considered superior to non-personalized protocol for individuals with this condition [26]. The CRP is a marker of inflammation and stress response in a human cell. It is secreted in the hepar by hepatocytes. It is well-known for its presence in cases of biological or chemical stressors such as an infection and a poisoning [27]. Furthermore, it is not only a marker of inflammation, but also an important molecule to regulate inflammatory processes [28]. Research has shown that not only could bioactive stressors such as infections be a reason to eject markers of inflammation, but also in the presence of prolonged stressors such as obesity [29]. That is why researchers are analyzing the presence of CRP in more subtle stressors that were not linked with cell stress response earlier. Another condition that can cause the secretion of a C-reactive protein is post-traumatic stress disorder since it can cause hormone dysregulation and alterations in inflammatory signaling [30].

## 5. Conclusions

This paper shows the positive impact of daily EEG neurofeedback intervention in analog mission astronauts. Further research should include more subjects in order to confirm the obtained trends and the statistical significance of the methods used. This paperindicates that differences in CRP are more related to the interaction between groups and time than to any single factor. It is necessary to explore this topic of reducing the stress response in isolated individuals because space mission companies will probably need well-adapted workers in the near future.

## Data Availability

The data used in this research paper are unavailable due to privacy reasons. In special cases, they could be made available after the subjects’ approval.

## Abbreviations

CRP	c-reactive protein
SI	social isolation
EEG	electroencephalography
